# Editorial: Language teacher psychology: New perspectives in multilingual contexts

**DOI:** 10.3389/fpsyg.2022.1109726

**Published:** 2022-12-13

**Authors:** Honggang Liu, Lawrence Jun Zhang, Vincent Greenier

**Affiliations:** ^1^School of Foreign Languages, Soochow University, Suzhou, China; ^2^Faculty of Education and Social Work, University of Auckland, Auckland, New Zealand; ^3^School of Language, Linguistics, Music and Visual Culture, University of Aberdeen, Aberdeen, United Kingdom

**Keywords:** language teacher psychology, multilingual contexts, new findings, multiple theoretical perspectives, multiple approaches

Language Teacher Psychology (LTP), which focuses on the individual differences between language teachers as a constituent of the language teacher education enterprise, deserves an in-depth investigation as a significant realm of research. It is a mature field now moving toward a diverse range of Research Topics, including socio-emotional factors, self-efficacy, and resilience, from the original focus on teacher cognition, identity, self-regulation, and motivation (Mercer and Kostoulas, 2018). Meanwhile, LTP is also explored *via* multiple theoretical perspectives, including the epistemological positions emanating from socio-psychological, sociocultural, socio-cognitive, and functionalist perspectives as well as from educational ecology, among others, to gain a deeper understanding of the complexity and dynamism of teachers' psychological profiles and behavioral practices in multilingual and multicultural settings. Against this background, the current Research Topic is timely, with 38 studies underpinned by a range of theoretical understandings, for example, control-value theory, social cognitive theory, complex dynamic systems theory, and ecological systems theory. The studies in this Research Topic have been conducted in diverse contexts, namely, China, Chile, New Zealand, Malaysia, Iran, and the United Kingdom.

In this editorial, we first offer an introduction to these 38 studies under six themes, with inspirations from the EMPATHICS^1^ model (Oxford, 2016) and Williams et al. (2016). Then we move to discuss the theoretical perspectives and research approaches utilized in these studies. Finally, we propose our recommendations for future studies in LTP.

## Diversified themes of language teacher psychology

The following six-themed research strands form this volume. They are teacher emotional factors (emotion, emotional labor, cognition, wellbeing), teacher motivational factors (flow, motivation, and meaningful engagement), teacher grit, teacher agency/adaptive expertise, teacher self-factors (self-efficacy, identity, and psychological empowerment), and teacher belief and perception.

### Teacher emotional factors

Teacher emotions are intimately connected to individual features and shaped by cultural and political contexts (Fried et al., 2015) in a complex and dynamic way, which are mirrored in the studies of this Research Topic. Sun and Yang find that Chinese senior high school English teacher emotion undergoes a U-shape-like change from positive to negative and then finally to a positive state in their dynamic interactions with the microsystem in which they work (e.g., students, family members, and friends), mesosystem (e.g., colleagues and administrators), exosystem (e.g., parents and organizations), and macrosystem (e.g., educational policy). Tao's quantitative investigation unveils Chinese teachers' intermediate levels of teacher mindfulness, work engagement, and classroom emotions. It unveils the role of engagement in mediating the effect of teacher mindfulness on classroom emotions.

Emotional labor has enjoyed wide popularity as a core topic in teacher emotion research. Four studies in this volume are around this topic regarding the specific features of emotional labor, influencing factors of emotional labor, and its relationship with other psychological variables (i.e., job satisfaction). The study of Bao et al. explores the Chinese as an additional language (CAL) teacher's emotional labor in a family-based context in New Zealand. It is found that this teacher has complex positive and negative emotional experiences, and her emotional labor strategies (i.e., surface acting and expression of naturally felt emotions) are highly associated with teacher identity and their relationships with students. Wang and Song's research finds three main influential factors for Chinese teacher emotional labor, which pertains to teachers' adaptation to online teaching technology, the invisibility of the online teaching space (the teaching context where teachers lack face-to-face communication), and the peripheral environment of the space (the situation where the noises occur and affect teacher emotion). Zhao et al.'s exploration into the English for Specific Purposes (ESP) teacher emotional labor indicates that the major emotional labor is closely associated with students' disengagement, specifically concerning students' low participation, misbehavior, absence, and non-completion of homework. Teachers experience mixed feelings of anger and empathy and display their capacity for resistance, negotiation, and reflection when they negotiate emotional labor to respond to the institutional rules. In Zhu and Zhou's study, they primarily unravel the correlation between English as a foreign language (EFL) teachers' job satisfaction and emotional labor subgroups, such as surface acting (SA), expression of naturally felt emotion (ENFE), and deep acting (DA). They find that teachers who predominantly rely on ENFE and DA report high levels of job satisfaction, and those with higher levels of SA experience lower levels of job satisfaction.

Teacher cognition is an emotional factor (Oxford, 2016; Zhang and Sun, 2022). Studying teacher cognition contributes to understanding what teachers think, believe, and know, facilitating their teaching practice and students' academic performance (Sun and Zhang, 2022; Zhao and Zhang, 2022). In this volume, some scholars attempt to investigate the multidimensionality of language teacher cognition and its relationship with teaching practice. Cao et al. demystify the multidimensional structure of English for Medical Purposes teacher cognition as teacher attitude, teacher belief, teacher learning, teacher support, role identification, and teacher practice *via* the exploratory factor analysis. Chen and Abdullah unveil the incongruence between EFL teacher cognitions and practices in the context of educational equity. They identify experiential (i.e., teachers' learning and professional experiences) and contextual (i.e., curriculum demand, teacher education programs, teacher authority, and exam-oriented doctrines) factors that bring about this mismatch. A survey by Gao and Yang reveals some key factors that facilitate EFL teacher's professional development from a novice teacher to a teacher leader, such as teaching experience, in-service training, administrative promotion, and self-reflection. This echoes well what has been reported in the literature about teacher learning, especially in this challenging time when the impact of COVID-19 on teaching and learning is so strongly felt (Gao and Zhang, 2020). Liu and Chen's research indicates that student-teachers have limited knowledge of genre pedagogies and face challenges in enacting genre-based writing instruction, such as the lack of professional expertise, the heavy workload, and the high-stakes testing culture.

Clear, strong, and well-documented evidence supports the close association between wellbeing and teacher emotion and practices in two studies of this special issue. Wang describes the rewarding experiences and challenges Chinese teachers encounter at the Confucius Institute in the United Kingdom. She further analyzes the effect of ecological factors (i.e., language-related issues, intercultural adjustment, teacher status, and work-life balance) on teacher wellbeing and some strategies for developing this wellbeing (i.e., seeking social support, deploying personal strengths, and establishing harmonious teacher-student relationships). Wang and Chen explore the increasing sense of wellbeing of English teachers working in unfavorable rural schools during a professional development program. They bring to light some influencing factors related to this increasing sense of wellbeing, for example, sufficient training content and support from experienced teacher educators.

### Teacher motivational factors

Regarding the letter M in the EMPATHICS model, motivation and motivational factors (i.e., flow) are addressed in the current collection, with much emphasis on its relationships with other psychological variables. Sobhanmanesh utilizes a quantitative approach to examine the correlation among Iranian EFL teacher flow state, personality traits, and emotional intelligence. The results show that emotional intelligence and personality traits (except agreeableness of the big five personality traits) have significant relationships with flow, and the conscientiousness and openness of personality traits are the strongest predictors of a flow state.

EFL teacher motivation has also been explored from different theoretical perspectives, where self-determination theory (SDT) is favored by three studies in this volume. Sato et al. adopt SDT and L2 motivational self-system to examine the relationship between teacher motivation and perceived burnout in Chile. They determine that teacher motivation negatively predicts their perceived burnout, but demotivators positively predict teachers' perceived burnout. Findings also affirm a weak correlation between teacher motivation and L2 motivation. Inspired by the SDT, Yang unpacks five motivational orientations of Chinese college EFL teachers toward their continuing professional development: cognitive interest and inner-directed academic improvement, academic self-fulfillment and obligation, academic and social responsibility, social recognition and promotion, and lacking the intention for continuing professional development. Zheng and Huan depict three motivational trajectories (i.e., accelerating trajectory, growing trajectory, and fluctuating trajectory) of Chinese EFL teachers and analyze the influencing factors for their motivational trajectories in the collaborative action research, including the psychological self (i.e., competence, autonomy, and relatedness) and contextual factors (i.e., classroom, school ethos, and the community of collaborative action research, and the national educational policies).

Teacher meaningful engagement is essential in promoting their professional development and students' academic achievements in the complex educational context (Oxford, 2016). In this Research Topic, research on factors that influence meaningful engagement in teaching and learning activities and their impact on student learning is given special attention. Kong et al. attempt to study EFL teacher engagement in culturally responsive teaching (CRT) and outline four types of teacher engagement related to knowledge and practice. They are “*xing er bu zhi* (teachers practice CRT well but do not know the concept well), *zhi er bu xing* (teachers know CRT but do not practice it), *bu zhi bu xing* (teachers neither know nor practice CRT), and *zhi xing he yi* (teachers know CRT and practice it well)” (p. 5). The authors also study the internal factors (i.e., teacher belief) and external factors (i.e., test-oriented culture) on teacher engagement. Wang J. et al. find that teacher engagement has a direct and positive effect on student's English achievement. They also reveal that it has indirect effects on English achievement in the online context through autonomous motivation and enjoyment as well as the chain mediating of autonomous motivation and positive academic emotions (enjoyment and relief).

### Teacher grit

Grit emerges as a topic in this volume as the alternative to perseverance (or passion) in the EMPATHICS model. In a mixed-methods study, Shabani et al. explore the differences in pedagogical thought categories (PTC) between high-grit and low-grit teachers through a grit scale and stimulated recall interviews, discovering significant differences between the two groups of teachers in the number and list of dominant pedagogical thought categories. Additionally, high-grit EFL teachers mainly possess dominant PTC involving language management, procedure check, affective, self-reflection, progress review, beliefs, and problem check, and low-grit teachers display the dominant PTC concerning language management, procedure check, time check, progress review, and problem check.

### Teacher agency/teacher's adaptive expertise

Exploring teacher agency is conducive to understanding how teachers exercise conscious efforts to navigate the influence of contextual realities, thus sustaining and strengthening their professional commitment and development (Tao and Gao, 2017). In this special issue, research from Gao and Cui, Gu et al., Huang and Yip, and Jiang et al. pave a new avenue for recognizing and perceiving teacher agency, with much emphasis on the enactment/exertion of teacher agency and its related influencing factors. Gao and Cui explore the changing affective profile of new teacher leaders in Taiwan and the intricate dynamic links among complex teacher emotional experience, agency, and power. The results show that leadership power can be affected by the teacher leader's individual agency, and the power relations change correspondingly affects their emotional experience in the educational reform. Gu et al. report that the EFL teacher exerts agency through passionate exploration of adaptive teaching and continuous investment in autonomous learning for his professional development in the under-resourced Chinese environment. They document a number of resources that trigger the formation and development of teacher agency, such as challenging working environments, past experiences, and long-term goals and passion for teaching. Huang and Yip propose a three-layered triadic reciprocity framework to delineate the complex process of teacher agency development, involving the degree of agency (i.e., proactive, reactive, and passive agency) and highlight the role of four properties of human agency, i.e., intentionality, forethought, self-reactiveness, and self-reflectiveness and the joint effect of personal, environmental, and behavioral determinants on exerting teacher professional agency. Jiang et al.'s findings show that Chinese EFL teachers display variability in exercising their professional agency in the formative assessment activities and unveil the impact of some demographic variables (i.e., gender, age, and professional title) and contextual factors (i.e., workload, physical and mental health, leadership, and scientific research pressure) on the enactment of teacher agency. In addition, inspired by the complex dynamic systems theory, Xiang et al. reveal the complexity of teachers' adaptive expertise and adaptive teaching practices in academic writing in light of non-linearity, interconnectedness, and self-organization features.

### Teacher self-factors

Inspired by Oxford's (2016) EMPATHICS and Williams et al.'s (2016) interpretation of the self, studies of teacher self-efficacy, identity, and psychological empowerment (similar term to self-efficacy) are grouped into the current theme.

Three empirical studies in this special issue develop around teacher self-efficacy, focusing on its structure, level, and relationship with other variables, such as teaching satisfaction and support, engagement, agency, and resilience. Liu et al. confirm a bi-factorial structure of the Chinese EFL teacher self-efficacy in livestream teaching in the Chinese context, namely, instructional self-efficacy and technological self-efficacy, and find a moderate-to-high level of teacher self-efficacy. Han et al. find the significant mediating role of self-efficacy in relationships between university EFL teachers' perceived teaching support, satisfaction, and teacher innovation in the online teaching environment. Han and Wang conclude that there are significant and positive relationships among EFL teacher self-efficacy, work engagement, and reflection, and their self-efficacy and work engagement significantly predict the reflection.

As a modern zeitgeist of sociological research, and certainly also a current and critical topic in teacher education, identity has been highly emphasized by the contributors to this Research Topic. Special attention is given to the construction, change, and role of teacher identity in different educational settings. Chen et al. point out that Chinese student-teacher emotion is determined and mediated by their professional identities. Chen and Huang describe the interaction of three sub-identities (trainer/educator, researcher, practitioner) of translation and interpreting (T&I) teachers and analyze the relationship between T&I teachers and the above three sub-roles to demystify the development of teacher identity. Jiang draws a trajectory of the change of teacher identity from English-for-general-purposes (EGP) teachers to English-for-specific-purposes (ESP) teachers during the curriculum reform in China. She finds that the substance of teacher identity is relatively stable, and the authority-source of teacher identity, the self-practice of teacher identity, and the telos (the ultimate goals and purpose) have been in a state of transition from EGP teachers to ESP teachers. Sun et al. investigate novice Chinese as a foreign language (CFL) teacher identity construction from positioning and affordance perspectives in New Zealand. Their results show that consistent self-positionings at the social, institutional, and individual levels positively affect teacher identity, and perceived affordance factors (i.e., training and classroom management), especially affordance as opportunities, benefit the construction of their identities of being CFL teachers. Wang R. et al. demonstrate the dynamic construction of a Chinese EFL pre-service teacher's language teaching identity during the whole practicum stage and emphasize the role of environmental needs, individual needs, and personal expectations in shaping teacher identity. Wang and He delve into the construction of EFL teacher professional identity in the university-school collaborative project in mainland China. The results showcase a spiral process of identity construction from “practitioner” to “researcher” to “practitioner,” and there are salient influencing factors, including teachers' research experience, curriculum reform, and communication in the community. Zhang and Wang investigate the change in English teacher professional identity under the influence of international professional development programs and teachers' negotiating process with the environment upon return to China. The findings reveal that teachers' international experiences significantly impact their professional identity development, motivating them to construct multiple identities as language teaching professionals, university academics, and change agents, and personal, interpersonal, and contextual factors influence the negotiating process of their identity.

Lei and Xu explore the under-researched self-variable of psychological empowerment, highlighting the individuals' expectations of their competence and sense of control, which to a large degree, shows the similarity with the notion of self-efficacy. They reveal a five-dimensional structure of college English teacher psychological empowerment concerning teacher perception and experience of work: meaningfulness, teaching autonomy, occupational competence, professional impact, and social status.

### Teacher belief and perception

Li et al. develop and validate a scale about pre-service CAL teacher belief and extract a tri-factorial structure of teacher beliefs, namely, beliefs about Chinese language teaching, beliefs about the Chinese language, and beliefs about Chinese language learners. Gao et al. investigate Chinese language teacher belief about challenges and solutions during the early stage of the pandemic and the development of their agency and resilience. Their findings reveal the significant relationship among perceived challenges and solutions based on quantitative analysis and highlight that teacher beliefs, agency, and resilience resonate through intrapersonal and interpersonal reflections and temporal and contextual affordances. In the wake of the implementation of global education in schools, teacher perception of global competence in English language teaching has drawn some attention. Yaccob et al. point out that ESL language teachers display positive perceptions of global competence in English language teaching and learning but express their negative perceptions of professional development programs organized by the Ministry of Education in Malaysia.

## Multiple theoretical perspectives and approaches to studying language teacher psychology

This Research Topic explores the interdisciplinary trend and diversified theoretical lens of language teacher psychology and promotes the development of research on LTP. The contributors of this volume have successfully utilized several perspectives to investigate LTP in a well-organized manner.

The psychological perspective, as the traditional perspective in researching LTP, is taken to view the structure of teacher psychology (e.g., the bi-factorial structure of the Chinese EFL teacher self-efficacy in online teaching by Liu et al.) and the relationship among teachers' psychological factors (e.g., the relationship among self-efficacy, work engagement, and reflections by Han and Wang). A social perspective on LTP emphasizes the change in teacher psychology in their dynamic interactions with social, cultural, or historical factors (e.g., the development of teacher beliefs, agency, and resilience in the pandemic and the broader environment by Gao et al.). A complex and dynamic perspective on LTP highlights the complexity, dynamics, non-linearity, interconnectedness, and self-organization of language teachers' psychological factors (i.e., wellbeing, motivation, and identity) in their teaching practices (Chen and Huang; Wang R. et al.; Xiang et al.; Zheng and Huan).

It is worth noting that the ecological perspective has grown in popularity and serves as an essential theoretical lens to explore LTP in a broader ecological context (Mercer, 2021; Liu et al., 2022), with its apparent potential to explain the interconnected factors of LTP, such as personal attributes or assets, relationships with family, friends, or leaders, and policy requirements. Research on LPT from this perspective focuses more on the LTP's development and changes in the specific context. It helps unveil the interaction between developing teachers and the environment where they survive and survive. The studies in this Research Topic reveal the dynamic interaction between individual psychological factors and different ecological systems by examining emotion (Sun and Yang) and agency (Gao and Cui; Jiang et al.) and providing suggestions for promoting language teachers' professional development.

Among the 38 studies, different theoretical perspectives have been viewed on LTP using a multitude of research methods in this special issue: quantitative (10), qualitative approach (19), mixed-methods (8), and synthetic review (1). The qualitative approach is highly favored in the Research Topic because it allows for a comprehensive thick description and articulation of the psychological characteristics of language teachers and provides a deeper understanding of their cognition, belief, and behavior in complex educational settings. Several data collection techniques have been employed in this Research Topics, such as questionnaires, interviews, narrative-framed journals, stimulated recall interviews, a tree of life, and reflective diaries. For example, in the study of Wang R. et al. a tree of life, as a newly-used self-reflective instrument, is commissioned to collect data about language teacher identity before, during, and after the teaching practicum.

## Recommendations for future studies of language teacher psychology

As the exploration of LTP continues to emerge, this special issue, with the joint efforts of the authors, proposes some significant recommendations that can be drawn on to promote the psychological development of language teachers from both macro and micro perspectives, and beyond, enabling teachers to maintain a better psychological state to manage the various challenges caused by the surrounding circumstances and people, as well as the organizations and institutions where they operate.

As for suggestions regarding the individual aspect, language teachers are encouraged to strengthen their inner drive, develop their responsiveness and openness, exercise their agency, and actively participate in learning communities. From the micro perspective, teacher leaders need to build rapport with teachers and motivate teachers to strengthen communication with integral stakeholders, such as colleagues, administrators, and their communities. From the macro perspective, there is an urgency for education policymakers to revisit education issues and reconsider the positioning of the English language in a given context. Governments have to provide financial support for teacher education, especially teacher professional development, reduce the financial disparities between different school sectors, and achieve equity in education. From the outset, the government and education administration should concentrate on the essential impact of language teachers' psychological characteristics and provide future and current educators with psychological development, career planning, guidance, professional advancement initiatives, and a complete evaluation mechanism. Finally, university and school leaders should establish a collaborative environment for teachers and focus on dynamic, situated, and meaningful professional development that centers on teacher learning and teacher wellbeing.

## Author contributions

HL: conceptualization, draft, and revision. LZ and VG: conceptualization and revision. All authors contributed to the article and approved the submitted version.

## Footnotes

^1^Based on the PERMA model (Seligman, 2011), Oxford (2016) proposed the EMPATHICS, a well-being model for foreign language learning. Letters in EMPATHICS stand for different psychological factors, namely, emotion and empathy (E), meaning and motivation (M), perseverance (P), agency and autonomy (A), time (T), hardiness and habits of mind (H), intelligence (I), character strengths (C), and self-factors (S).
